# Editorial: Therapies for brain injury

**DOI:** 10.3389/fphar.2024.1445516

**Published:** 2024-06-21

**Authors:** Luigi Manni, Marzia Soligo, Antonio Chiaretti, Leon Morales-Quezada

**Affiliations:** ^1^ Institute of Translational Pharmacology—CNR, Rome, Italy; ^2^ Department of Women’s Health Sciences, Fondazione Policlinico Universitario A. Gemelli—IRCCS, Rome, Italy; ^3^ Spaulding Research Institute, Spaulding Rehabilitation Hospital, Charlestown, MA, United States

**Keywords:** brain injury, neuropharmacology, brain plasticity, neuroinflammation, network diffusion model

Brain injuries pose significant challenges to both neurology and medical science and represent a significant social burden due to the resulting lifelong functional deficits and disabilities. The current therapeutic strategies often fall short in preventing the ominous progression of brain damage. However, researchers are making remarkable strides in developing innovative therapies to address these issues.

Traumatic brain injury (TBI) typically involves two aspects: primary brain injury, which occurs at the trauma site, and secondary brain injury, mediated by associated pathological processes like ischemia, hypoxia, lipid peroxidation, neuroinflammation, mitochondrial dysfunction, and oxidative stress. While primary brain injury can often be treated surgically, secondary brain injury remains a therapeutic focus to protect nerve damage and control neuroinflammation. Despite no existing drug completely repairing damaged nerves, controlling neuroinflammation could offer a therapeutic window to reduce progressive brain damage and improve nerve function. Here, Zhao et al. review the use of pleiotropic drugs targeting neuroinflammation. The concept of pleiotropic neuroprotection embraces multi-target therapeutic approaches to mitigate the complex neuroinflammatory responses and secondary brain injuries. Various neuroprotective drugs, including progesterone, statins, erythropoietin, polyphenolic compounds, minocycline, glitazones and amantadine have shown promise in preclinical studies but have often failed to demonstrate significant clinical benefits in humans. These failures can be blamed to limitations in existing animal models, insufficient pharmacokinetic and pharmacodynamic studies, as well as to differences between preclinical and clinical trial methodologies.

Among emerging and promising therapeutic compounds, cannabidiol (CBD) has shown neuroprotective properties that could potentially counteract the damaging effects of secondary brain injury by restoring neurotransmitter balance post-injury, reducing neuroinflammation and microglia activation, maintaining blood-brain barrier integrity and regulating cerebral blood flow, and enhancing neurogenesis. In their contribution, Aychman et al. review the literature on the topic, indicating pre-clinical research in support of CBD’s potential to improve outcomes for brain injured patients, but posing that systematic human clinical trials are needed to verify its efficacy and determine optimal dosing strategies. However, although the data on the potential of CBD in the therapy of TBI currently only comes from pre-clinical studies, the multiplicity of pathological mechanisms that this active ingredient appears to be able to target make it a good candidate for future pharmaceutical development.

In their original research, part of this Research Topic, Hiskens et al. provide data indicating that acetyl-L-carnitine (ALC) treatment mitigates neurodegeneration, neuroinflammation, and cognitive deficits in a mouse model of repetitive mild traumatic brain injury (rmTBI). ALC reduces the expression of key neurodegenerative and inflammatory markers such as microtubule associated protein tau (MAPT), glial fibrillary acidic protein (GFAP), allograft Inflammatory Factor 1 (AIF1) and tumor necrosis factor (TNF), particularly in the cortex and hippocampus. The neuroprotective effects of ALC are evidenced by improved performance in neurological severity scores and spatial learning and memory tasks. ALC’s therapeutic potential is highlighted by its ability to cross the blood-brain barrier and its safety profile, making it a promising candidate for long-term administration in TBI contexts.

The cerebrovascular system constitutes another potential target for pharmacological interventions aimed at containing secondary damage from trauma. A rat model of TBI was used by Al Yacoub et al. to investigate the therapeutic potential of SB-612111, a synthetic antagonist of the nociceptin/orphanin FQ peptide (NOP) receptor, the fourth member of the opioid receptor superfamily. Mild TBI increased levels of nociceptin/orphanin FQ (N/OFQ) in cerebrospinal fluid (CSF), which correlated with decreased cerebral blood flow (CBF), and this effect was mitigated by SB-612111. The study observed that TBI activated ERK and cofilin-1 within 3 h post-injury, with ERK activation correlating with increased CSF N/OFQ levels. The findings suggest that targeting the N/OFQ-NOP receptor system could be a potential therapeutic strategy for treating cerebrovascular dysregulation following TBI.

Early neuroprotective pharmacotherapeutic intervention may be crucial in the management of brain injuries, with the aim of quickly dampening the neuro-damaging sequelae ultimately leading to the development of disabilities. Mansour et al. propose a systematic review of randomized controlled studies, examining the effects of early adjunctive pharmacotherapy on serum levels of brain injury biomarkers in patients with traumatic brain injury (TBI). The review included 11 studies that investigated the impact of various pharmacotherapeutic interventions on biomarkers such as neuron-specific enolase (NSE), glial fibrillary acidic protein (GFAP), and S100 beta (S100 ß). The results suggest that tetracyclines, metformin, and memantine may be promising candidates for improving neurological outcomes in TBI patients, based on their effects on serum biomarker levels. However, the review also identifies several limitations and gaps in the current evidence, such as heterogeneity in biomarker monitoring protocols, confounding factors, and the need for standardized measurement protocols.

Computational tools may enhance our understanding of brain injury, guide therapeutic strategies, and accelerate research progress. They may, among others, contribute to develop biomechanical and neural network models, empower network pharmacology and pharmacological target screening, provide optimal integration of clinical, genetic and imaging data, in order to extract meaningful insights, and also support clinical decision by predictive and risk assessment tools. In this Research Topic, Vergni et al. re-examines the dynamics of network diffusion and introduces reaction-diffusion models on networks to better describe degenerative brain dynamics. Numerical simulations illustrate that different reaction terms and initial conditions can lead to vastly different outcomes, highlighting the versatility of reaction-diffusion models. The new models allow for non-constant diffusion rates and varied reaction terms, providing a detailed and adaptable framework for studying brain pathologies. The reaction-diffusion models can be tailored to specific brain diseases by adjusting model parameters and initial conditions, making them potentially valuable tools for predicting disease progression and evaluating treatment efficacy.

In conclusion, the pursuit of effective therapies for brain injury remains a critical mission—one that holds the potential to improve lives, reduce social burden, and alleviate suffering. The integration of respective competence among neurologists, neuroscientists, pharmacologists, and bioinformaticians is essential to unlock new possibilities and pave the way toward better outcomes for those affected by brain injuries.

